# Supplementary material for: SpaMOAL is a deep learning method that enables accurate spatial domain identification from multi-omics data
Source: PLoS Biol. 2026 Sep 16;24(9):e3003690. doi: 10.1371/journal.pbio.3003690 (PMC13623151; doi:10.1371/journal.pbio.3003690)
Supplement: Supplementary file 1 [file pbio.3003690.s001.docx]

**Supplementary Text**

1. **Simulation data generation**

To provide evaluation data with ground truth, we generated seven multi-modal datasets. Six were created using scMultiSim, and the remaining one was generated by SpatialGlue, following the approach outlined by Townes et al.. Specifically, we first used scMultiSim to generate three RNA-ATAC dual-modal datasets, and then produced three image-integrated RNA-ATAC triple-modal datasets by incorporating simulated H&E images. scMultiSim enables simultaneous modeling of cell identity, gene regulatory networks, cell-cell interactions, and chromatin accessibility, while also introducing technical noise. We generated datasets with different hierarchical structures by varying configurations such as gene regulatory networks, cell numbers, gene counts, and random seeds, detailed configurations are provided in Supplementary Table S4. As scMultiSim does not support the generation of triple-omics data, we directly adopted the RNA-ATAC-protein dataset generated by SpatialGlue. Each data type was modeled using a tailored statistical distribution, the transcriptomic data were modeled with a zero-inflated negative binomial distribution to accurately represent their high sparsity and over-dispersion characteristics. Both proteomic data and chromatin accessibility measurements were modeled using a standard negative binomial distribution, which is appropriate for characterizing their count-based data features.

2. **Ablation studies**

To evaluate the contribution of each loss function to SpaMOAL's performance, we conducted ablation studies on the Simulation1 dataset. As shown in Supplementary Table S5, when the model lacks the matching loss ($\mathcal{L}_{mat}$), correlation loss ($\mathcal{L}_{cor}$), reconstruction loss ($\mathcal{L}_{rec}$) or contrastive loss ($\mathcal{L}_{con}$), its performance degrades to varying degrees. This demonstrates the positive contributions of each of the aforementioned components to the model's performance. Furthermore, to fully demonstrate the advantages and versatility of SpaMOAL, we have supplemented extensive benchmark comparisons and additional ablation experiments. We incorporated representative state-of-the-art unimodal and bimodal methods, including STAGATE (single-modal spatial transcriptomics method) and SpaGCN^1^ (spatial coordinate + histology image integration method), to comprehensively verify the superiority of our three-modal fusion strategy. We also performed ablation experiments under incomplete modal input settings and have released the corresponding code on GitHub. The results on multiple labeled datasets demonstrate that SpaMOAL is not limited to fully paired multi-modal data and can stably perform spatial domain identification under both single-modal and dual-modal conditions, greatly improving model versatility and applicability. All newly added results are presented in revised Supplementary Figures S3, S5, S8, and S9. Consistent results on both simulated and real biological datasets show that SpaMOAL achieves more accurate and spatially coherent domain segmentation than STAGATE and SpaGCN. In addition, we further compared the performance of different histological feature encoders, including conventional ImageNet-pretrained CNNs and the advanced pathology-specific foundation model UNI. As shown in revised Supplementary Figure S6, histology-specific foundation models produced modest improvements over ImageNet-pretrained CNNs for spatial domain identification, although the gains were limited.

3. **Sensitivity to parameters**

We evaluated the sensitivity of SpaMOAL to its key hyperparameters—the loss coefficients α, β, and λ—using simulated datasets. The default values were set to α = 0.8, β = 0.5, and λ = 0.9. To assess the effect of varying a single parameter, we held two parameters constant at their defaults while adjusting the third. As shown in Supplementary Fig. S4, although variations in these parameters did influence performance, the overall effect was modest, indicating that SpaMOAL is robust to a range of hyperparameter values.

4. **Detailed parameter settings**
(1) SpaMOAL: The model was trained using the Adam optimizer with a learning rate of 0.001. We applied a dropout rate of 0.1 and set the temperature coefficient for contrastive learning to 0.5. For reproducibility, the random seed was fixed at 0, and spatial clustering was performed using the mclust algorithm. All parameters used have been supplemented on GitHub.
(2) SpatialGlue: The random seed was set to 2022. To ensure a fair comparison, the same number of clusters was used across all methods for each dataset with mclust employed for domain identification.
(3) MISO: We configured MISO with a random seed of 100, setting both ind_views and combs to "all". Clustering was conducted via the K-means algorithm, with the cluster counts matched to those of SpaMOAL.
(4) STAGATE: The rad_cutoff parameter was tailored to specific datasets: 500 for the human breast cancer dataset, 0.2 for Simulation 2, 1 for other simulated datasets, and 2 for all remaining datasets. Spatial domains were identified using mclust with cluster numbers consistent with SpaMOAL.
(5) SpaGCN: SpaGCN was implemented with a learning rate of 0.5 and s=1, using a random seed of 100. The beta parameter was set to 20 for the human breast cancer dataset, 30 for mouse embryonic datasets, and 40 for both mouse brain multi-omics and simulated datasets. The number of clusters remained consistent with the SpaMOAL configuration.
